# Alterations in anatomic and functional imaging parameters with repeated FDG PET-CT and MRI during radiotherapy for head and neck cancer: a pilot study

**DOI:** 10.1186/s12885-015-1154-8

**Published:** 2015-03-17

**Authors:** Manil Subesinghe, Andrew F Scarsbrook, Steven Sourbron, Daniel J Wilson, Garry McDermott, Richard Speight, Neil Roberts, Brendan Carey, Roan Forrester, Sandeep Vijaya Gopal, Jonathan R Sykes, Robin JD Prestwich

**Affiliations:** 1 Department of Nuclear Medicine, St. James’ University Hospital, Leeds Teaching Hospitals NHS Trust, Leeds, UK; 2 Department of Clinical Radiology, St. James’ University Hospital, Leeds Teaching Hospitals NHS Trust, Leeds, UK; 3 Division of Medical Physics, University of Leeds, Leeds, UK; 4 Department of Medical Physics, St. James’ University Hospital, Leeds Teaching Hospitals NHS Trust, Leeds, UK; 5 Department of Radiotherapy Physics, St. James’ University Hospital, Leeds Teaching Hospitals NHS Trust, Leeds, UK; 6 Department of Radiotherapy, St. James’ University Hospital, Leeds Teaching Hospitals NHS Trust, Leeds, UK; 7 Department of Clinical Oncology, St. James’ University Hospital, Leeds Teaching Hospitals NHS Trust, Leeds, UK; 8 St. James’ Institute of Oncology, Level 4 Bexley Wing, Beckett Street, Leeds, LS9 7TF UK

**Keywords:** Head and neck neoplasms, Radiotherapy, Computed tomography, Fluorodeoxyglucose F18, Positron-emission tomography, Magnetic resonance imaging

## Abstract

**Background:**

The use of imaging to implement on-treatment adaptation of radiotherapy is a promising paradigm but current data on imaging changes during radiotherapy is limited. This is a hypothesis-generating pilot study to examine the changes on multi-modality anatomic and functional imaging during (chemo)radiotherapy treatment for head and neck squamous cell carcinoma (HNSCC).

**Methods:**

Eight patients with locally advanced HNSCC underwent imaging including computed tomography (CT), Fluorine-18 fluorodeoxyglucose (FDG) positron emission tomography (PET)-CT and magnetic resonance imaging (MRI) (including diffusion weighted (DW) and dynamic contrast enhanced (DCE)) at baseline and during (chemo)radiotherapy treatment (after fractions 11 and 21). Regions of interest (ROI) were drawn around the primary tumour at baseline and during treatment. Imaging parameters included gross tumour volume (GTV) assessment, SUV_max_, mean ADC value and DCE-MRI parameters including Plasma Flow (PF). On treatment changes and correlations between these parameters were analysed using a Wilcoxon rank sum test and Pearson’s linear correlation coefficient respectively. A p-value <0.05 was considered statistically significant.

**Results:**

Statistically significant reductions in GTV-CT, GTV-MRI and GTV-DW were observed between all imaging timepoints during radiotherapy. Changes in GTV-PET during radiotherapy were heterogeneous and non-significant. Significant changes in SUV_max_, mean ADC value, Plasma Flow and Plasma Volume were observed between the baseline and the fraction 11 timepoint, whilst only changes in SUV_max_ between baseline and the fraction 21 timepoint were statistically significant. Significant correlations were observed between multiple imaging parameters, both anatomical and functional; 20 correlations between baseline to the fraction 11 timepoint; 12 correlations between baseline and the fraction 21 timepoints; and 4 correlations between the fraction 11 and fraction 21 timepoints.

**Conclusions:**

Multi-modality imaging during radiotherapy treatment demonstrates early changes (by fraction 11) in both anatomic and functional imaging parameters. All functional imaging modalities are potentially complementary and should be considered in combination to provide multi-parametric tumour assessment, to guide potential treatment adaptation strategies.

**Trial Registration:**

ISRCTN Registry: ISRCTN34165059. Registered 2nd February 2015.

## Background

The use of radiotherapy ± chemotherapy is now established as a standard of care in the management of locally advanced head and neck squamous cell carcinoma (HNSCC), both for unresectable disease [1] and organ preservation [2]. Intensity modulated radiotherapy (IMRT) has been widely adopted for the treatment of HNSCC [3]. IMRT along with image guided radiotherapy (IGRT) can provide a highly conformal dose distribution with steep dose gradients, sparing critical adjacent organs at risk.

Despite the increasing complexity and high degree of conformality of modern radiotherapy techniques, radiation therapy is routinely planned on a pre-treatment ‘planning’ computed tomography (CT) scan acquired at a single timepoint. A further concept is that of adaptive radiotherapy, which takes into account patient and/or tumour changes which occur during treatment [4]. Treatment modifications are commonly only made in the event of on-treatment problems such as significant weight loss or mask fitting problems. However, it is recognized that tumours respond variably during a course of fractionated radiotherapy [5]. An assessment of this response to treatment may allow a timely individualization of treatment. For example, if on-treatment imaging was an accurate response prediction tool, imaging changes could be used to guide dose escalation in the event of an inadequate early response [6], or a de-intensification of therapy in light of a favorable early response in order to maximize therapeutic ratio. Future clinical trials are likely to increasingly test adaptive approaches individualising therapy.

In order to develop adaptive radiotherapy strategies, imaging biomarkers are needed to determine prognostically significant early tumour changes during treatment. Computed tomography (CT) remains the mainstay of radiotherapy planning, providing accurate geometrical data along with electron density maps to allow dose calculation. However, low soft tissue resolution and dental artifacts hinder tumour delineation with CT, as shown by wide inter-observer variability in contouring head and neck tumours on planning CT scans [7]. Anatomic magnetic resonance imaging (MRI) sequences provide excellent soft tissue contrast, and can reduce inter-observer variability in target contouring [8,9]. Functional imaging may offer information on factors which influence treatment outcomes, e.g. tumour cellularity, perfusion, hypoxia. Fluorine-18 fluorodeoxyglucose (FDG) positron emission tomography (PET) is the most widely used functional imaging modality in head and neck cancer and is commonly combined with CT (PET-CT) providing additional biological information about tumours complementary to anatomic imaging [9,10]. FDG is a widely used radiolabelled glucose analogue taken up by metabolically active cells. Functional MRI sequences also provide biological tumour information. Diffusion weighted MRI (DW-MRI) relies upon the free and random diffusion of water molecules with restricted diffusion occurring in highly cellular areas of a tumour; the degree of diffusion restriction is quantified by the apparent diffusion coefficient (ADC) value. Baseline DW-MRI has been shown to predict local control of HNSCC [11]. Dynamic contrast enhanced MRI (DCE-MRI) provides a signal which is related to the underlying perfusion and permeability of the tumour microenvironment. DCE-MRI characteristics have been found to be predictive of short term treatment responses [12,13]. Current available data regarding imaging changes during radiotherapy are limited [14]. Important questions arise with regard to i) which is the most suitable imaging modality to assess early response during treatment, and ii) what is the optimal timing of on-treatment imaging assessments to guide adaptive radiotherapy strategies.

In this hypothesis generating pilot study, we aim to examine on-treatment changes occurring on CT, FDG PET-CT and MRI including DW-MRI and DCE-MRI sequences in the primary tumour which may potentially guide selection of imaging modality and timing for response assessment studies.

## Methods

### Inclusion criteria

Inclusion criteria for this prospective single centre pilot study were as follows: age ≥18 years old, histologically proven squamous cell carcinoma of the head and neck region, WHO performance status 0-2, decision to proceed with (chemo)radiotherapy with curative intent following discussion in a multi-disciplinary meeting, measurable primary cancer on routine pre-treatment imaging (CT and/or MRI), and provision of fully informed consent. Patients were excluded from the study if there was poorly controlled diabetes, contraindication to MRI or an estimated glomerular filtration rate <30 ml/min/1.73 m^2^. This study was approved by the Research Ethics Committee (National Research Ethics Committee Yorkshire and the Humber-Bradford, 11/YH/0212) and Administration of Radioactive Substances Advisory Committee (ARSAC).

### Treatment

All patients underwent 70 Gy of radiotherapy delivered in 35 once daily fractions delivered over a period of 7 weeks as per departmental protocol. Treatment was delivered using a 5-7 field step-and-shoot IMRT technique. Standard concurrent chemotherapy was cisplatin at a dose of 100 mg/m^2^ on days 1 and 29. Cetuximab was delivered if cisplatin was contraindicated, at a dose of 400 mg/m^2^ on day -7 and then weekly at a dose of 250 mg/m^2^ during radiotherapy.

### Imaging schedule

The imaging schedule was performed as part of the clinical study. Baseline imaging consisted of FDG PET-CT and MRI scans. Repeat FDG PET-CT and MRI scans during radiotherapy were performed +/- 3 days of delivering fractions 11 and 21, which were approximately 2 and 4 weeks from the commencement of radiotherapy, respectively.

### Image acquisition

#### FDG PET-CT

FDG PET-CT imaging was performed on a 64-section GE Discovery 690 PET-CT system (GE Healthcare, Amersham, UK). Baseline half-body PET acquisition with a dedicated head and neck acquisition (3-4 bed positions, 2 minutes per bed position) from skull vertex to carina was performed 60 minutes following a 400 MBq injection of intravenous FDG. The CT component of the head and neck acquisition was obtained after a 25 second delay following a bolus of 100 mls of iodinated contrast (Niopam 300, Bracco Ltd, High Wycombe, UK) injected at 3 ml/s using the following settings; 120 kV, variable mA (min 10, max 600, noise index 12.2), tube rotation 0.5 s per rotation, pitch 0.969 with a 2.5 mm section reconstruction. The contrast-enhanced CT component of the PET-CT scan, acquired with a 5-point thermoplastic radiotherapy immobilization mask fitted and room laser alignment, was also used for radiotherapy planning according to routine clinical protocols. The remainder of the PET acquisition from symphysis menti to upper thighs was acquired following this with a delayed post-contrast CT component using similar scan acquisition parameters and a contiguous 3.5 mm reconstruction.

During radiotherapy, only a dedicated head and neck PET acquisition was performed with an accompanying contrast-enhanced CT component using the same PET and CT imaging parameters detailed above.

### MRI

Images were acquired on a 1.5 T Siemens Magnetom Avanto (Siemens Healthcare, Erlangen, Germany). The following sequences were acquired in the standard diagnostic position using a dedicated head/neck coil; single shot EPI diffusion-weighted images (b = 0, 400 and 800 s/mm^2^, TR = 6200 ms, TE = 89 ms, 40 x 4 mm thick slices with a 1 mm slice gap, acquired voxel size = 1.2 x 1.2 x 4.0 mm voxel), 3D spoiled gradient echo dynamic contrast enhanced scan (TR = 3.23 ms, TE = 0.93 ms, flip angle =21°, 40 x 5 mm slices, 2.5 s temporal resolution, 150 time points, acquired voxel size = 2.4 x 1.8 x 7.1 mm), axial post-contrast T1-weighted spin echo image (TR = 831 ms, TE = 8.6 ms, 105 x 2 mm thick contiguous slices, acquired voxel size = 0.9 x 0.9 x 2.0 mm). The patient was repositioned in the radiotherapy immobilization device and the axial post-contrast T1-weighted image was repeated as well as a fat saturated T2-weighted scan (TR = 4430 ms, TE = 76 ms, voxel size = 0.8 x 0.7 x 3.0 mm). A contrast agent (0.2 ml/kg Dotarem, Guerbet, France, 3 ml/sec) was injected after approximately 10 measurements of the 3D spoiled gradient echo sequence.

### Image analysis

In each imaging modality, assessment of the primary tumour was carried out as detailed below by a single experienced head and neck radiologist (MS 6 years of experience).

### FDG PET-CT

Image analysis was undertaken on a dedicated PET workstation (Advantage Windows, version 4.5, GE Healthcare, Amersham, UK). The maximum tumour standardized uptake value (SUV_max_) was derived by drawing a region of interest (ROI) encompassing the primary tumour, which defined the gross tumour volume (GTV) on PET. This was achieved by using an adaptive thresholding technique, known as the Homburg algorithm [15], calculated from the mean primary tumour SUV (SUV_mean_) when applying a 70% of SUV_max_ isocontour, the background tissue SUV_mean_ and two scanner specific coefficients (determined from phantom studies).

### CT, MRI

Image analysis was undertaken using XD3 software (Mirada Medical, Oxford, UK). GTV-CT was defined as the volume of enhancing tumour, whilst the GTV-MRI was defined as the area of high signal representing tumour on the T2-weighted image using the T1-weighted images for anatomic cross reference. DW-MRI analysis was undertaken on a Leonardo workstation (Siemens Healthcare, Erlangen, Germany). Analysis consisted of visual contouring of the area of restricted diffusion within the primary tumour on the b800 images, using both the T1- and T2-weighted images for anatomic cross reference, to calculate the GTV-DW. These contours were applied to the accompanying apparent diffusion coefficient (ADC) maps, calculated from the single shot EPI sequence, and a mean ADC value was calculated for each ROI.

DCE-MRI analysis was undertaken using validated in-house software, PMI 0.4 [16]. An arterial input function was measured by selecting the single brightest pixel in the internal carotid artery on a map of the maximal signal enhancement. A plasma flow map was calculated by deconvolution and the entire primary tumour was visually outlined on this map using the T1- and T2-weighted images for anatomic cross reference. Tissue concentration-time curves in the primary tumour were fitted to a two compartment exchange model, producing functional DCE-MRI parameters including Plasma Flow (PF), Plasma Volume (PV), Interstitial Volume (also known as Extravascular Extracellular Space, ν_e_), Permeability Surface Area Product (PS), Extraction Fraction (EF) and K^trans^ each of which reflect different physiologic parameters within the tumour microenvironment [17]. All concentrations were approximated by subtraction of the baseline signal.

### Statistical analysis

Patient characteristics were recorded at baseline. Percent change in multi-parametric measurements occurring during treatment were analysed using a Wilcoxon rank sum test using the Statistics Toolbox of Matlab R2013b with the null hypothesis that the median percentage change is zero. Correlations between parameters were performed using Pearson’s linear correlation coefficient, also in Matlab R2013b. A p-value of < 0.05 was considered statistically significant.

## Results

Eight patients entered the study between November 2011 and June 2012. All completed treatment with a median follow up of 24 months (range 13-28). Patient demographics and tumour characteristics are shown in Table 1. Seven patients were treated with concurrent cisplatin; one patient (patient 4) received concurrent cetuximab due to deafness contra-indicating cisplatin. All patients completed treatment with 70 Gy in 35 fractions over 7 weeks of radiotherapy. On follow up, 7 of 8 patients are disease free. One patient (patient 7) relapsed with brain metastases with loco-regional control.

**Table 1:** **Patient demographics and tumor characteristics**

Patient	Primary tumor site	T-stage	N-stage	Differentiation	GTV_MR_(cm^3^)	Follow-up (months)	Disease recurrence
1	Oropharynx, tonsil	2	2b	Poorly	3.0	28	No
2	Supraglottis, Epiglottis	3	2b	Well	14.7	27	No
3	Hypopharynx, pyriform fossa	3	0	Moderately	4.0	28	No
4	Oropharynx, base of tongue	4a	1	Poorly	32.8	25	No
5	Oropharynx, base of tongue	2	1	Poorly	10.6	24	No
6	Oropharynx, base of tongue	2	2b	Moderately	6.4	20	No
7	Oropharynx, base of tongue	2	2b	Poorly	16.7	13	Distant metastases
8	Oropharynx, base of tongue	1	2b	Poorly	2.0	21	No

**Figure 1:**
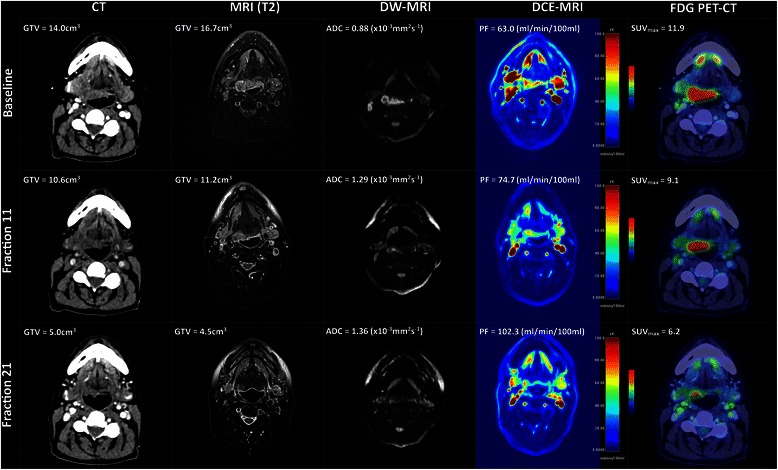
**Multi-modality imaging changes during radiotherapy.** A case of a patient with a poorly differentiated squamous cell carcinoma of the base of tongue, T2N2bM0 treated with concurrent chemoradiotherapy to a dose of 70 Gy in 35 fractions over 7 weeks with concurrent cisplatin 100 mg/m^2^ days 1 and day 29. Imaging was acquired at baseline, fraction 11 and fraction 21 timepoints. Representative axial images at each timepoint are shown, illustrating CT, T2-weighted MRI, DW-MRI, DCE-MRI, and FDG PET-CT images. Colourwash panels show intensity of FDG uptake and PF.

All patients completed imaging with FDG PET-CT and MRI at baseline and at the fraction 11 timepoint. One patient (patient 1) did not undergo further imaging at the fraction 21 timepoint due to treatment-related toxicity. One patient (patient 2) did not undergo MRI at the fraction 21 timepoint due to an MRI scanner technical error. Six patients completed all imaging as planned within the study. Baseline FDG PET-CT was performed a median of 19 days pre-treatment (range 13-24). Baseline MRI was performed a median of 8 days (range 2-16) pre-treatment. FDG PET-CT and MRI at the fraction 11 timepoint took place at a median of -0.5 days (range -1 to +3) and 0 days (range -1 to +2) from fraction 11 respectively. FDG PET-CT and MRI at the fraction 21 timepoint took place at a median of +1 (range -1 to +3) and +2 days (range -2 to +4) from fraction 21 timepoint respectively. Representative multi-modality images from one patient (patient 7) are shown in Figure 1. During analysis, the GTV was not identifiable on the CT for patient 1 due to dental amalgam. SUV_max_ measurement was inaccurate due to high blood glucose on serial imaging for patient 1 (data not shown). All other images acquired were suitable for interpretation.

**Figure 2:**
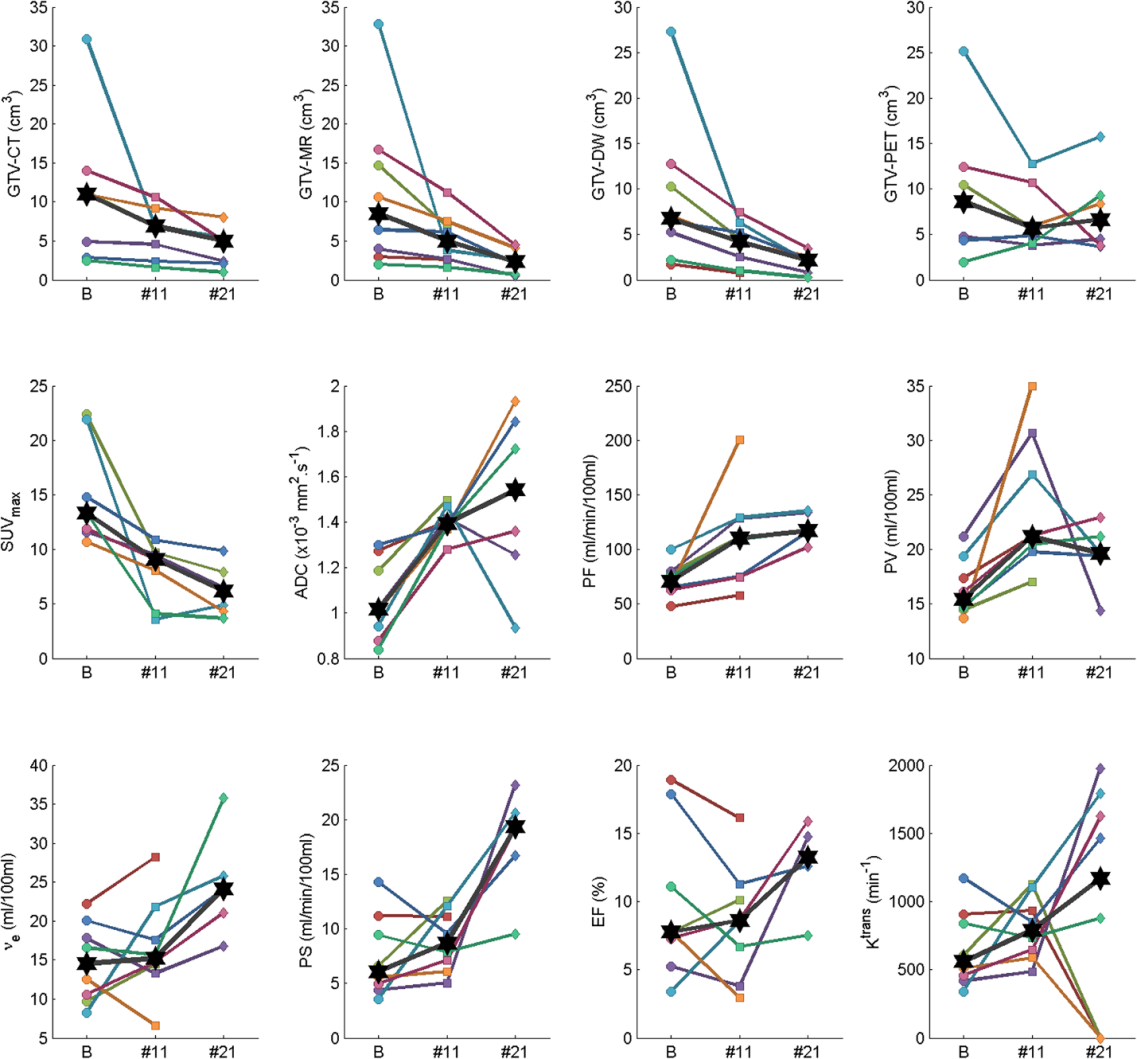
**Absolute changes in anatomical and functional imaging parameters during radiotherapy.** Plots of GTV-CT, GTV-MRI, GTV-DW, GTV-PET, SUV_max_, mean ADC value (ADC), Plasma Flow (PF), Plasma Volume (PV), Interstitial Volume (ν_e_), Permeability Surface Area Product (PS), Extraction Fraction (EF) and K^trans^ at baseline (B), fraction 11 (#11) and fraction 21 (#21) timepoints. ✶= median data point at each imaging timepoint. Coloured lines represent individual patients.

**Figure 3:**
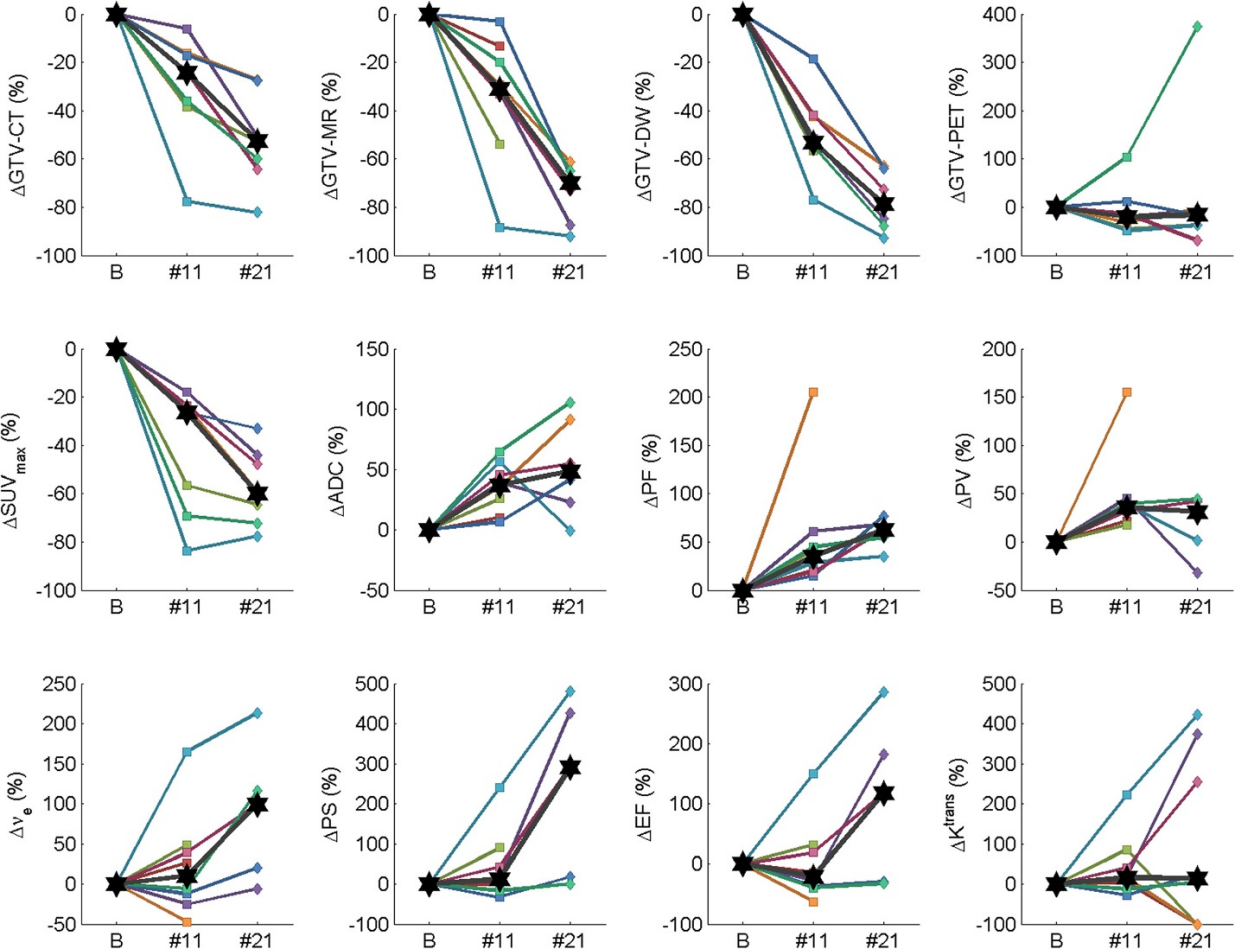
**Percentage changes in anatomical and functional imaging parameters during radiotherapy.** Plots of percentage change in GTV-CT, GTV-MRI, GTV-DW, GTV-PET, SUV_max_, mean ADC value (ADC), Plasma Flow (PF), Plasma Volume (PV), Interstitial Volume (ν_e_), Permeability Surface Area Product (PS), Extraction Fraction (EF) and K^trans^ at baseline (B), fraction 11 (#11) and fraction 21 (#21) timepoints. ✶= median percentage change at each imaging timepoint. Coloured lines represent individual patients.

The anatomic primary tumour volumes as contoured on CT, MRI and DW-MRI (GTV-CT, GTV-MRI and GTV-DW respectively) at serial timepoints, progressively reduced to varying degrees during treatment for all patients (Figure 2). Statistically significant percentage reductions in multi-modality anatomic primary tumour volumes (Wilcoxon Ranked Sum, p < 0.05, no correction for repeated measures) were observed between all imaging timepoints (Figure 3, Table 2). GTV-PET showed a decrease in 5 patients between baseline and the fraction 11 timepoints, but a paradoxical increase in 5 patients between the fraction 11 and fraction 21 timepoints; these percentage changes in metabolic tumour volume did not reach statistical significance.

**Table 2:** **Median [range] and p-value of percentage change in parameters between imaging timepoints (Baseline, Fraction 11 and Fraction 21)**

Parameter	Baseline to Fraction 11	Baseline to Fraction 21	Fraction 11 to Fraction 21
GTV-CT	**−24 [−78,−6]**	**−53 [−82,−27]**	**−23 [−53,−13]**
(cm^3^)	**p = 0.016**	**p = 0.016**	**p = 0.016**
GTV-MR	**−31 [−88,−3]**	**−70 [−92,−61]**	**−58 [−81,−32]**
(cm^3^)	**p = 0.008**	**p = 0.031**	**p = 0.031**
GTV-DW	**−53 [−77,−19]**	**−79 [−93,−63]**	**−62 [−73,−36]**
(cm^3^)	**p = 0.008**	**p = 0.031**	**p = 0.031**
GTV-PET	−20 [−49,105]	−16 [−69,374]	18 [−64,132]
(cm^3^)	p = 0.375	p = 0.297	p = 0.578
SUV_max_	**−26 [−84,−18]**	**−60 [−78,−33]**	−19 [−47,36]
	**p = 0.016**	**p = 0.016**	p = 0.219
Mean ADC value	**37 [6,64]**	48 [−1,105]	16 [−37,41]
(x10^−3^mm^2^s^−1^)	**p = 0.008**	p = 0.063	p = 0.563
Plasma Flow (ml/min/100 ml)	**35 [15,205]**	62 [35,77]	7 [4,54]
	**p = 0.008**	p = 0.063	p = 0.063
Plasma Volume	**36 [18,155]**	31 [−32,45]	−2 [−53,8]
(ml/100 ml)	**p = 0.008**	p = 0.313	p = 0.625
Interstitial Volume	11 [−47,165]	99 [−6,213]	37 [18,129]
(ml/100 ml)	p = 0.461	p = 0.125	p = 0.063
Permeability Surface Area Product (ml/min/100 ml)	12 [−33,240]	290 [1,481]	75 [21,360]
	p = 0.313	p = 0.063	p = 0.063
Extraction Fraction (%)	−21 [−62,150]	118 [−32,286]	55 [12,290]
	p = 0.641	p = 0.313	p = 0.063
K^trans^ (min^−1^)	16 [−28,222]	15 [−100,422]	41 [−100,307]
	p = 0.148	p = 0.461	p = 0.742

Statistically significant results indicated in bold type.

The multi-parametric functional measurements showed varied changes at serial timepoints during radiotherapy (Figure 2). SUV_max_ decreased from baseline to fraction 11 in all patients and fell further at the fraction 21 timepoint in 6 of 7 patients; the percentage change between baseline and the fraction 11 and fraction 21 timepoints was statistically significant (p = 0.016). The mean ADC value increased from baseline to the fraction 11 timepoint in all patients and showed a further increase at the fraction 21 timepoint in 4 of 6 patients; the percentage change between baseline and the fraction 11 timepoint was statistically significant (p = 0.008). Plasma Flow progressively increased in all patients at fraction 11 compared with baseline; 5 of 6 patients showed a further increase at the fraction 21 timepoint., However, only percentage changes in Plasma Flow and Plasma Volume between baseline and the fraction 11 timepoint reached statistical significance (p = 0.0078, p = 0.0078), whilst percentage changes in ν_e_, EF, PS and K^trans^ during radiotherapy did not reach statistical significance (Figure 3, Table 2).

There were several parameters that showed a significant correlation between the percentage change (Δ) from baseline to the fraction 11 timepoint; a full listing of parameter pairs with significant correlation is given in Table 3. ΔGTV-CT was correlated with ΔGTV-MRI and ΔGTV-MRI was correlated with ΔGTV-PET. However, ΔGTV-CT was not correlated with ΔGTV-PET and ΔGTV-DW was inconsistently correlated with only ΔGTV-MRI between the baseline and fraction 11 timepoint and ΔGTV-CT between the fraction 11 and fraction 21 timepoints. There were also negative correlations between both ΔGTV-CT and ΔGTV-MRI with some of the DCE parameters (ΔK^trans^, ΔPS, ΔEF, Δν_e_) and a positive correlation with ΔSUV_max_. Strong positive correlations were observed between some of the DCE parameters. For instance ΔK^trans^ had a near perfect correlation with ΔPS.

**Table 3:** **Statistically significant (p < 0.05) correlations between percentage changes (**Δ**) in pairs of measured volumes (GTV-CT, GTV-MR, GTV-DW, GTV-PET) and functional parameters (SUV**_max_**, mean ADC value (ADC), Plasma Flow (PF), Plasma Volume (PV), Interstitial Volume (**ν_e_**), Permeability Surface Area Product (PS), Extraction Fraction (EF) and K**^trans^**)**

Time interval	Parameter 1	Parameter 2	Correlation coefficient	p-value
**Baseline to Fraction 11**	ΔGTV-CT	ΔGTV-MR	0.820	0.0239
	ΔGTV-CT	Δν_e_	−0.923	0.0030
	ΔGTV-CT	ΔPS	−0.874	0.0101
	ΔGTV-CT	ΔEF	−0.885	0.0081
	ΔGTV-CT	ΔK^trans^	−0.872	0.0105
	ΔGTV-CT	ΔSUV_max_	0.910	0.0044
	ΔGTV-MR	ΔGTV-DW	0.785	0.0210
	ΔGTV-MR	Δν_e_	−0.817	0.0134
	ΔGTV-MR	ΔPS	−0.973	0.0001
	ΔGTV-MR	ΔEF	−0.887	0.0033
	ΔGTV-MR	Δ K^trans^	−0.974	0.0000
	ΔGTV-DW	ΔPS	−0.753	0.0311
	ΔGTV-DW	Δ K^trans^	−0.754	0.0306
	ΔPF	ΔPV	0.969	0.0001
	Δν_e_	ΔPS	0.914	0.0015
	Δν_e_	ΔEF	0.983	0.0000
	Δν_e_	Δ K^trans^	0.909	0.0018
	ΔPS	ΔEF	0.960	0.0002
	ΔPS	Δ K^trans^	1.000	0.0000
	ΔEF	Δ K^trans^	0.955	0.0002
**Baseline to Fraction 21**	ΔGTV-CT	ΔGTV-DW	0.851	0.0316
	ΔGTV-CT	ΔPF	0.926	0.0237
	ΔGTV-MR	ΔADC	0.887	0.0185
	ΔGTV-MR	ΔPS	−0.954	0.0118
	ΔGTV-MR	ΔEF	−0.962	0.0088
	ΔGTV-MR	Δ K^trans^	−0.964	0.0019
	ΔADC	Δ K^trans^	−0.847	0.0334
	ΔPF	Δν_e_	−0.936	0.0191
	ΔPF	ΔSUV_max_	0.934	0.0203
	ΔPS	ΔEF	0.981	0.0032
	ΔPS	Δ K^trans^	1.000	0.0000
	ΔEF	Δ K^trans^	0.981	0.0030
**Fraction 11 to Fraction 21**	ΔGTV-DW	ΔPF	0.899	0.0381
	ΔPS	ΔEF	0.972	0.0057
	ΔPS	Δ K^trans^	1.000	0.0000
	ΔEF	Δ K^trans^	0.967	0.0073

## Discussion

Adaptive radiotherapy planning for HNSCC is a very attractive goal to allow the early individualization of treatment. Modern imaging techniques now offer the opportunity to track anatomic and/or functional tumour alterations during treatment. These imaging modalities are candidates to provide an early response assessment, which may be used to individually tailor treatment strategy. This adaption could potentially take the form of intensification or de-intensification of treatment based upon early response. On-treatment imaging could also be used to guide dose delivery, for example being used to plan a radiotherapy boost.

With regard to anatomic imaging modalities, mean anatomic volumes were reduced by > 30% at fraction 11 and > 50% by fraction 21. Cao et al. [18] in a study of 14 patients reported a 28% reduction in tumour volume after two weeks of treatment in those with locally controlled disease. Dirix et al. [19] in a study of 15 patients with various head and neck cancers (including 6 oropharyngeal cancers) found an approximate halving of tumour size after 4 weeks of radiotherapy, as assessed by CT and MRI. Geets et al. [20] studied 18 patients with pharyngo-laryngeal cancers, finding significant reductions in tumour size on CT and MRI following 46 Gy of treatment. These consistent findings of substantial reductions in tumour size during treatment in a range of head and neck tumour sites emphasizes the opportunity for treatment strategies based around early treatment responses.

The implementation of functional imaging techniques to assess tumour response during treatment remains uncertain. GTV-PET showed an initial reduction at the fraction 11 timepoint in 5 patients but then a paradoxical increase in the same number of patients at the fraction 21 timepoint. This was related to confounding peri-tumoural inflammation and reducing tumour to background ratio resulting in difficulties in applying automated segmentation algorithms to contour metabolic tumour volumes, which has been described previously [21]. Moule et al. [22,23] reported on the use of serial FDG PET in a series of 12 patients; SUV_max_ values were found to progressively reduce during treatment. Background SUV_max_ was not found to alter significantly with radiation dose, but because tumour uptake dropped, thresholding methods were found to be unreliable in segmenting tumour from background [22,23]. Therefore these observations regarding GTV-PET are likely due to limitations of segmentation algorithms rather than reflecting the underlying biological processes. As shown in Figures 2 and 3 and Table 2, SUV_max_ was found to consistently fall with significant reductions in SUV_max_ from baseline observed during treatment. These findings are consistent with other studies investigating on-treatment FDG PET imaging [20,22-24]. Hentschel et al. [24] have reported the largest series of 37 patients who underwent serial FDG PET imaging at baseline and at end of 1st or 2nd week (after 10 Gy or 20 Gy), 3rd or 4th week, and 5th or 6th week of radiotherapy. A >50% reduction in SUV_max_ on FDG PET acquired after 10 Gy or 20 Gy (n = 8 of 37) was found to correlate with 2 year disease free and overall survival. By contrast with our results with FDG PET at fraction 21, the authors commented that it was commonly not possible to determine SUV_max_ following 30-40 Gy of treatment due to therapy-associated peri-tumoural inflammation.

Significant changes in mean ADC value were observed during treatment (Figure 2, Figure 3 and Table 2). The observed increase in ADC during treatment reflects reduced tumor cellularity and hence a likely response to treatment. These findings are consistent with 3 prior studies examining DW-MRI as a predictive imaging modality during chemoradiotherapy [19,25,26]. In the study of 30 patients by Vandecaveye et al. [25], the change in ADC value was predictive of 2 year loco-regional control. Similarly, Kim et al. [26] of 40 patients, reported an increase in ADC values measured on imaging one week into a course of chemoradiotherapy to predict a complete treatment response. Dirix et al. [19] previously showed that tumour volume contoured on diffusion imaging reduced in volume during treatment; in addition, and as we have found, tumour volume on diffusion imaging appeared smaller than on anatomic MRI throughout the study.

Only very limited data is available on DCE-MRI changes during radiotherapy in the literature. In our cohort of 8 patients, significant alterations in Plasma Flow and Plasma Volume were observed during treatment (Figure 2, Figure 3 and Table 2). Cao et al. [18] similarly observed an increase in Plasma Flow after 2 weeks of radiotherapy. Plasma Flow is regarded as a key parameter in the context of radiotherapy and has been shown to have a negative correlation with the degree of tumour hypoxia [27]. Therefore the observed increases in plasma flow during treatment may correlate with improved perfusion, reduced hypoxia and consequentially reduced radioresistance. By contrast, patterns of alterations in the commonly reported functional parameter K^trans^ were inconsistent. Dirix et al. [19] examined the use of DCE-MRI during treatment and did not find useful information on disease response. The very high correlations between K^trans^ and PS found in this study are indicative of high plasma flow compared to PS. This suggests that the uptake of contrast is limited by the permeability of the vessels rather than in-flow.

One key question to guide future studies is which imaging modality or combination of techniques should be used to provide early response prediction. Multiple correlations were observed between both anatomic and functional imaging parameters (Table 3) but it remains unclear as to which combination is optimal. Some imaging techniques are not widely available and are more difficult to implement into routine clinical practice. A limited number of studies to date have examined the value of on-treatment imaging as an early predictor of outcome. Changes on early on-treatment imaging with FDG PET [24] and FLT PET [28] have been shown to correlate with disease outcomes. The data presented here confirms that marked changes occur early during treatment in both anatomic and functional imaging. In terms of percentage changes compared with baseline, no single imaging modality appears superior. Our data is limited by its small sample size and loco-regional disease control within the treatment field in all patients, both of which preclude any useful correlation with outcome. However, from these data, anatomic imaging with CT or MRI, or functional data derived from FDG PET, DW- or DCE-MRI are all candidate imaging modalities to investigate early response predictors. Decisions on which imaging parameters are most likely to be clinically valuable will depend to a certain extent upon the availability and logistics of imaging. The advent of combined PET/MR scanners may be valuable in advancing these multimodality imaging approaches, allowing acquisition of multiple modalities at one scan session.

Adoption of an adaptive treatment strategy requires the availability of prognostic information as early as possible during treatment. Image acquisition after fraction 11 and fraction 21 of radiotherapy was aimed at identification of a potential imaging timepoint upon which further exploratory studies looking at prognostic value of imaging biomarkers be based upon. Marked changes occur early during treatment in both anatomic and functional imaging readouts, although the magnitude of change between fraction 11 and 21 timepoints was generally less than that seen at fraction 11 compared with baseline. An earlier timepoint during treatment provides more opportunity to allow treatment adaption. Therefore, these results suggest that imaging after around two weeks of treatment is the most suitable timepoint to investigate in future studies examining treatment adaptation.

There are several limitations to this study. Patient numbers are small, and in particular two patients did not complete all planned imaging at the fraction 21 timepoint. This will have restricted the ability of the data to demonstrate significant associations in imaging changes from baseline and fraction 11 to fraction 21. A further possible limitation of this analysis is the method by which ROIs were constructed on functional imaging modalities. Limitations in FDG PET based tumour contouring during treatment are detailed above and the optimal method of segmenting PET imaging to define the tumour edge remains uncertain and controversial [29]. ROIs for DW-MRI and DCE-MRI were created with visual cross-reference to T1- and T2- weighted imaging but geometric distortions are known to preclude the current use of DW-MRI for tumour delineation for radiotherapy planning [30]. An alternative method using spatial co-registration of imaging modalities may have enabled more accurate construction and reproducible regions of interest. However, even with this methodology, there are potential errors in co-registration and uncertainties in which imaging modality most accurately reflects tumour volumes [31,32]. We adopted a pragmatic approach that would be readily applicable to clinical practice, although ongoing work is examining the spatial correlation of on-treatment multi-modality imaging changes.

## Conclusion

In summary, significant alterations with anatomic and functional imaging of the primary tumour were observed early (by fraction 11) in treatment. Significant but variable correlations between different imaging modalities existed. Each of these imaging modalities, either alone or in combination, remains a candidate to provide an early biomarker of outcome. The study confirms the potential of multi-parametric tumour assessment during radiotherapy to guide treatment adaptation strategies. Future studies will need to correlate each modality alone or in combination with outcome, to determine their relative value as imaging biomarkers to guide treatment individualization and adaption.

## Abbreviations

HNSCC: Head and neck squamous cell cancer
IMRT: Intensity modulated radiotherapy
IGRT: Image guided radiotherapy
CT: Computed tomography
FDG: Fluorine-18 fluorodeoxyglucose
PET: Positron emission tomography
MRI: Magnetic resonance imaging
DW: Diffusion weighted
DCE: Dynamic contrast enhanced
ARSAC: Administration of radioactive substances advisory committee
SUV_max_: Maximum standardized uptake value
ROI: Region of interest
GTV: Gross tumour volume
SUV_mean_: Mean standardized uptake value
ADC: Apparent diffusion coefficient
PF: Plasma flow
PV: Plasma volume
ν_e_: Extravascular extracellular space
PS: Permeability surface area product
EF: Extraction fraction
